# Short Tandem Repeat Genotyping of Medically Important Fungi: A Comprehensive Review of a Powerful Tool with Extensive Future Potential

**DOI:** 10.1007/s11046-024-00877-8

**Published:** 2024-08-03

**Authors:** Bram Spruijtenburg, Jacques F. Meis, Paul E. Verweij, Theun de Groot, Eelco F. J. Meijer

**Affiliations:** 1 Radboudumc-CWZ Center of Expertise for Mycology, Nijmegen, The Netherlands; 2 Canisius-Wilhelmina Hospital (CWZ)/Dicoon, Nijmegen, The Netherlands; 3 Cologne Excellence Cluster On Cellular Stress Responses in Aging-Associated Diseases (CECAD) and Excellence Center for Medical Mycology, Institute of Translational Research, University of Cologne, Cologne, Germany; 4 Department of Medical Microbiology, Radboud University Medical Center, Nijmegen, The Netherlands

**Keywords:** Short tandem repeats, Genotyping, Outbreak investigation, *Candida*, *Aspergillus*, Whole genome sequencing

## Abstract

Fungal infections pose an increasing threat to public health. New pathogens and changing epidemiology are a pronounced risk for nosocomial outbreaks. To investigate clonal transmission between patients and trace the source, genotyping is required. In the last decades, various typing assays have been developed and applied to different medically important fungal species. While these different typing methods will be briefly discussed, this review will focus on the development and application of short tandem repeat (STR) genotyping. This method relies on the amplification and comparison of highly variable STR markers between isolates. For most common fungal pathogens, STR schemes were developed and compared to other methods, like multilocus sequence typing (MLST), amplified fragment length polymorphism (AFLP) and whole genome sequencing (WGS) single nucleotide polymorphism (SNP) analysis. The pros and cons of STR typing as compared to the other methods are discussed, as well as the requirements for the development of a solid STR typing assay. The resolution of STR typing, in general, is higher than MLST and AFLP, with WGS SNP analysis being the gold standard when it comes to resolution. Although most modern laboratories are capable to perform STR typing, little progress has been made to standardize typing schemes. Allelic ladders, as developed for *Aspergillus fumigatus,* facilitate the comparison of STR results between laboratories and develop global typing databases. Overall, STR genotyping is an extremely powerful tool, often complimentary to whole genome sequencing. Crucial details for STR assay development, its applications and merit are discussed in this review.

## Introduction

Fungal infections have been increasing over the last decades, with the latest estimate of 2.5 million annual deaths [1]. Due to the opportunistic nature of a few hundred fungal species, these infections mainly take place in healthcare settings, with immunocompromised patients being most at risk for invasive infections [2]. The advance of medical care and treatments are accompanied by an increase in the number of immunocompromised patients, thereby increasing the prevalence of invasive fungal diseases. Although most fungal species thrive at temperatures below the human body temperature, climate change and natural disasters make fungi more thermotolerant, increasing the virulence of various fungal species [3]. In addition to the increased prevalence in healthcare settings, drug resistance is on the rise, often induced by medical or agricultural use of antifungal drugs, complicating diagnosis and treatment, and increasing mortality [4]. To address the urge of awareness of fungal disease, the WHO published the Fungal Priority Pathogens List [5]. Two fungi classified in the critical priority group include *Aspergillus fumigatus* and *Candida auris*. The first is a filamentous saprobic fungus ubiquitous in the environment and thermotolerant [6]. Due to the extensive use of azole pesticides in agricultural settings, this species is acquiring resistance on a large scale, with > 10% azole resistance reported in various studies and associated with an increased mortality rate [6]. Another emerging fungal species is the yeast *C. auris* which is often involved in nosocomial outbreaks and is rapidly acquiring antifungal resistance to multiple antifungal classes, thereby reducing treatment options and increasing mortality [7].

Given this emergence of drug resistant pathogens, the healthcare sector faces new threats. Hospitals need to treat an increasing number of patients, often immunocompromised and deal with new or uncommon pathogens, possibly elicited by prophylaxis or poorly understood risk factors [8]. Especially in resource-limited countries, hospitals are often unable to deal with the high number of admitted patients, resulting in insufficient infection prevention measurements, which may lead to nosocomial transmissions and infection outbreaks [9]. Environmental exposure is another source of fungal outbreaks [10]. To investigate or prevent nosocomial transmission during outbreaks, genotyping is of paramount importance and should be available at least for mycology reference laboratories [11]. Genotyping also allows investigations towards the spread and species population structure, leading to novel insights about the emergence of species and the spread of antifungal resistance. For example, *C. auris* genotyping demonstrated that this species was independently introduced at least six times in the human population and transmission of azole-resistant isolates is common [7]. Even echinocandin and pan-resistant isolates are reported, albeit rare [12]. This highlights the necessity and benefit of genotyping methods.

The current review discusses the application of short tandem repeat (STR) genotyping on various medically important fungal species and compares STR genotyping to other commonly employed methods. Although several fungal genotyping reviews are available, a comprehensive fungal STR review discussing all genotyped species is absent to date. First, all common genotyping methods currently in use will be presented, and their characteristics, advantages and limitations discussed. Next, we outline all STR genotyping schemes of various medically important fungal species and include a comparison to other methods, and conclude with future research directions and outlook. STR schemes have also been applied to numerous fungal plant pathogens and species closely related to fungi such as *Enterocytozoon bieneusi* [13, 14], but these are outside the scope of this review. Additionally, while we discuss important considerations when developing STR assays, the actual development of a STR assay is not elaborated further as it was previously discussed [11].

## Fungal Genotyping Methods

### Multilocus Sequence Typing

The multilocus sequence typing (MLST) method has been developed for several fungal species and has been used in numerous laboratories [15]. With this method several housekeeping genes are amplified with PCR, followed by Sanger sequencing. The generated sequences are then compared between isolates to determine the phylogenetic relatedness. As this method usually involves housekeeping genes, normally present in the pangenome, the typing scheme can be applied to a wide variety of isolates and is not hampered by mismatches, insertions or deletions in primer binding regions [16]. Another significant advantage is the excellent reproducibility of this method, even between different laboratories as incorrect base calling in Sanger sequencing is rare, although the calling of heterozygous bases in diploid fungi can be challenging. With MLST, the population structure of a species (e.g. clades or drug resistant lineages) can be determined and compared to a global database [17]. Using MLST, species complexes were elucidated and reclassified into distinct species, later confirmed by whole genome sequencing (WGS). For example, group 2 and 3 in the *Candida parapsilosis* complex were reclassified as *C. orthopsilosis* and *C. metapsilosis*, respectively [18]. Furthermore, with MLST the geographic origin of fungi has been determined, e.g. European *Cryptococcus gattii* species complex infections appeared partially autochthonous [19]. Although MLST had a vast contribution to phylogenetic studies, its use in outbreak settings is limited, as the few housekeeping genes, with their relatively low mutation rate, do not provide a high discriminatory power. Isolates with an identical sequence type (ST) may be epidemiologically unrelated and originate from a different source. The turnaround time and costs is another drawback, especially in resource limited countries.

### Amplified Fragment Length Polymorphism Analysis

One of the least labor intensive and most inexpensive genotyping methods is amplified fragment length polymorphism analysis (AFLP) DNA fingerprinting [20]. When executing this method, total genomic DNA is enzymatically digested and adapters are ligated, followed by selective PCR amplification. Next, gel electrophoresis is performed on the amplicons and the fingerprints can be compared to infer the genetic relatedness [21]. Isolates are differentiated by insertions or deletions within amplicons or polymorphisms in the restriction sites. Although a high resolution can be achieved in a single run, results between runs and especially between laboratories cannot be compared reliably [21, 22], which likely contributes to the reduced application of this method over the last years.

### Fourier-Transform Infrared Spectroscopy

In contrast to the assays above, Fourier-transform infrared (FTIR) spectroscopy does not involve PCR and is a high-throughput and straightforward technique [23]. The microbial cell composition generates an IR spectrum based on vibrational modes of light-absorbing bonds, primarily carbohydrates. The spectra of multiple isolates can be compared between each other to determine a possible genetic relationship. While the method is more often applied to bacteria, the usage in the field of medical mycology is limited and still in development [23]. To date, FTIR utilizing the IR Biotyper platform has been applied mainly to a few *Candida* species [24, 25].

### Whole Genome Sequencing

Currently, whole genome sequencing (WGS) is considered as the gold standard for fungal genotyping as it outcompetes the other methods in regards to resolution [26]. Isolates can be differentiated if these harbor a minimal genetic difference of few single nucleotide polymorphisms (SNPs), making this the ideal discriminatory power in outbreak settings [26]. However, the usage is restricted by several factors, including the associated costs, especially in resource-limited regions, and long turnaround time, which can be unfavorable when dealing with potential outbreaks. Moreover, WGS requires a high performance computational infrastructure to process the vast amount of generated data and sufficient bioinformatics capacity, while SNP calling pipelines require validation and standardization which is often lacking. Nonetheless, costs are steadily decreasing, as is the turnaround time.

### Short Tandem Repeat Genotyping

**Fig. 1:**
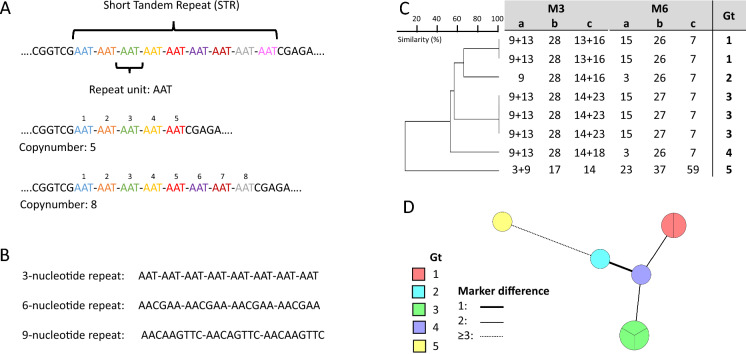
Short tandem repeat (STR) genotyping schematic. **A** Structure of STR and copy numbers, **B** different nucleotide repeat units, **C** dendrogram with corresponding copy number of eight diploid isolates, **D** minimum spanning tree of the same eight diploid isolates with the size of circles reflecting the number of isolates displaying an identical genotype

Considering the limitations of the previously mentioned genotyping techniques, short tandem repeat (STR) typing remains an appealing alternative method. STR genotyping assays rely on PCR amplification of a set of STR markers, also known as microsatellites or simple sequence repeats (SSRs) of which the length is subsequently determined with (gel) electrophoresis (Fig. 1) [11]. Depending on the ploidy of a given species, one or more copy numbers are generated and the comparison of copy numbers between isolates determines their genetic relatedness. The capacity of a single STR marker or the complete set of markers to distinguish isolates based on their genotype is defined as the discriminatory index, which can be calculated with the Simpson’s Diversity Index or the Hunter Index [27, 28]. Each STR marker exhibits a different discriminatory index, e.g. the number of found genotypes compared to the number of included isolates, with markers consisting of long repeat units generally displaying a lower variability than markers with short repeat units [29, 30]. A high resolution STR assay can be achieved by including a sufficient number of STR markers with each a high discriminatory index, making sure that unrelated isolates are differentiated from each other. For most STR schemes of fungi and other microorganisms, six to nine markers are included. The use of six to nine markers in fungal STR schemes makes it feasible to genotype large collections of isolates with a relatively high resolution as compared to other genotyping methods such as AFLP or MLST. Moreover, with AFLP it is impossible to add data prospectively. Yet, STR assays do not reach the resolution as obtained with WGS SNP analysis. Therefore, in STR scheme development and outbreak analyses where STR genotypes are similar, WGS should be used complimentary. Novel techniques based on next generation sequencing (NGS) might allow inclusion of more STR markers more easily, as will be discussed later.

The advantage of STR analysis above WGS SNP analysis is its preferred, more realistic timeframe. After DNA extraction, a single PCR run of 1–2 h is needed, followed by (gel) electrophoresis that typically takes less than one hour. A disadvantage of STR assays is the necessity to develop a new scheme for each species studied. The development of an STR scheme requires several important aspects. First, the scheme should have a high resolution to obtain a detailed population structure and differentiate isolates that are not closely related. Second, the primer binding site at the STR flanking sequences should be conserved so all genotypes within the species will be amplified. Third, STR markers should represent single copy targets to avoid multiple copy numbers for one marker. Fourth, it should be easy to apply and interpret the STR, which can be reached by selecting a small set of STR markers that show few stutter peaks and are easily amplified in multiplex, thereby also reducing costs [31]. Fifth, a validation by WGS should be performed, including both related and unrelated isolates to estimate the discriminatory power. Lastly and sixth, loci should be equally distributed across the genome.

## *Candida* and Other Medically Relevant Yeasts

### *Candida albicans*

Globally, the diploid yeast *C. albicans* is the most common causative agent of candidemia. It is the first ranking *Candida* species listed on the WHO Fungal Priority Pathogens List and considered to be among the most virulent species, while antifungal resistance is rare [32]. The yeast is part of the human microbiome and usually colonizes the oral cavity and gastrointestinal tract [33]. When the immune system of patients weakens, it is thought that *C. albicans* translocates to the bloodstream from non-sterile sites such as the gastrointestinal system [32]. Botterel et al*.* [34] developed an STR scheme that consists of three markers, identified in a single reference genome, and is amplified in monoplex. Application of this scheme included isolates from non-sterile sites and blood, and were often found to display identical STR genotypes, supporting an in-host source for the bloodstream infection (Table 1) [34, 35]. Another scheme was established by Sampaio et al*.*, [27].which includes ten markers, also amplified in monoplex and identified in a single reference genome. Typing results of this scheme were compared to WGS and isolates with the same STR genotype were also highly related according to WGS analysis [36]. As *C. albicans* is the primary cause of vulvovaginal candidiasis (VVC), the assay of Sampaio et al*.* [27] was used to type sequential isolates of patients with recurrent VVC. Most sequential isolates demonstrated the same STR genotype, suggesting a persistent vulvovaginal infection with the same strain, which was also found for sequential candidemia isolates [36]. WGS SNP analysis on the latter isolates also demonstrated a low genetic diversity, underscoring the concordance between WGS and STR results [36]. The two STR schemes developed for the diploid *C. albicans* amplify a lower number of markers when compared to most haploid species. Although the discriminatory power increases when more markers are amplified, diploid species generate up to two copy numbers per marker, while haploid species only generate one. As such, a lower number of markers can be utilized for diploid species to achieve a discriminatory power comparable to haploid species.

**Table 1:** Overview of short tandem repeat (STR) schemes developed for yeast species. For each scheme, the number of developed assays, amplified markers and validation with whole genome sequencing (WGS) is specified

Species	No. of assays	No. of markers	PCR characteristic	Isolate application	WGS validation (no. of isolates)	References
*Candida albicans*	2	3	Monoplex	French	No	[34]
		10	Monoplex	Portuguese	Yes (13)	[27]
*Candida auris*	1	15	Multiplex (5)	Global	Yes (196)	[30, 38]
*Candida parapsilosis*	2	3	Multiplex (1)	Mainly Portuguese	No	[49]
		6	Multiplex (2)	Global	Yes (15)	[50]
*Candida tropicalis*	3	6	Monoplex	Chinese	No	[56]
		8	Monoplex	Chinese	No	[58]
		6	Multiplex (2)	Global	Yes (42)	[57, 60]
*Candida glabrata* (*Nakaseomyces glabratus*)	1	8	Monoplex	Global	No	[63]
*Candida krusei* (*Pichia kudriavzevii*)	2	8	Monoplex	Chinese	No	[68]
		6	Multiplex (2)	Global	Yes (10)	[28]
*Wickerhamomyces anomalus* (*Candida pelliculosa*)	1	6	Multiplex (2)	Mainly Indian	Yes (11)	[73]
*Candida orthopsilosis*	1	4	Monoplex	Chinese	No	[76]
*Kodamaea ohmeri* (*Pichia ohmeri*)	1	3	Monoplex	Chinese	No	[79]
*Diutina catenulata* (*Candida catenulata*)	1	4	Monoplex	Mainly European	No	[81]
*Cryptococcus neoformans*	2	3	Monoplex	Global	No	[84]
		9	Multiplex (3)	Dutch, Cuban, Slovenian and Indian	No	[86, 88, 89, 91]
*Cryptococcus deneoformans*	1	7	Monoplex	Dutch and Slovenian	No	[88, 91]
*Cryptococcus deuterogattii*	1	10	Multiplex (3) and monoplex	Global	No	[93]

### *Candida auris*

As mentioned above, the haploid species *C. auris* is often involved in nosocomial transmission and various genotyping studies have been conducted [37]. A single STR assay has been developed to date, which in first instance consisted of four multiplex PCR reactions of three STR markers each (Table 1) [30]. This scheme of in total 12 STR markers was first applied to more than 400 isolates from numerous countries and revealed four major clades as previously found by other genotyping methods [30]. In a follow-up study the STR assay was validated and further optimized using a collection of 171 isolates previously analyzed by WGS SNP calling [38]. After addition of two additional STR markers to improve the discriminatory power for clade IV, all isolates differing more than 50 SNPs were distinguished by the extended STR scheme of 14 markers. While this is a relatively high resolution, it is not sufficient to define clonal transmission, as we previously found that sequential isolates from the same host obtained 11 SNPs at most in ten months [12]. Furthermore, STR genotypes within clades often demonstrate identical or highly related genotypes, while these are highly variable between clades. This is also observed with WGS SNP analysis, which demonstrated at least 10.000–200.000 SNPs difference between the currently identified six clades, while the genetic diversity within these clades is lower [39]. Thus, using this STR assay in a high prevalence region, identical STR profiles are insufficient to rule out clonal transmission and requires WGS SNP analysis to confirm nosocomial transmission. On the other hand, difference of a single STR marker does not rule out a clonal relationship, as within a collection of 171 isolates, there were two isolates that were identical according to WGS SNP analysis, but differed one copy number in one marker by STR analysis [38]. A different copy number of at least two markers was required to define isolates as unrelated in hospital outbreak analyses. Despite the suboptimal resolution as compared to WGS SNP analysis, the STR assay has a superior discriminatory power when compared to other methods. Using ITS sequencing, not all *C. auris* clades can be differentiated, yielding a lower discriminatory power compared to STR typing [25]. AFLP typing often groups *C. auris* isolates randomly or incorrectly while STR adequately demonstrates relatedness [25]. Additionally, MALDI-TOF MS and FTIR showed a poor correlation with STR results, indicating both methods needs to be improved before they can be applied to clinical epidemiological settings [25]. Thus, STR analysis is the genotyping tool of choice if WGS SNP calling is not applicable.

There are numerous examples of *C. auris* STR typing and mostly cover resource limited countries. The most notable application was the indication of a novel clade as a single isolate from Iran was not related to the other four clades [30]. The distinct clade V was subsequently confirmed by including more cases from Iran and the application of WGS SNP calling [41]. Additionally clade VI isolates were differentiated from prior reported clades via an in silico STR analysis on WGS data [40]. The high resolution and reproducibility of the STR assay also led to novel insights with its application on *C. auris* isolates from Brazil [42]. Here, STR assigned these isolates to clade I while inclusion of previously typed isolates allowed the identification of a distinct subclade, not found previously. This suggested these isolates were not introduced from countries with a high clade I prevalence like India but originated from a local source in Brazil [42].

Besides the investigation of population structures, the STR assay has been utilized for different *C. auris* outbreaks settings, as illustrated by the following two examples. In Kuwait, *C. auris* candidemia and colonization in patients from different hospitals were reported for 18 months [43]. Most isolates originated from a secondary-care hospital, and all these isolates, except one, were found to have an identical STR profile. As the single isolate also lacked the *ERG11*^*Y132F*^ mutation, in contrast to all other isolates, it was likely not related. In the other hospitals in Kuwait, slightly different STR genotypes were found, suggesting potential clonal transmission within the secondary-care hospital, however this was not confirmed by WGS SNP analysis [43]. The latter is essential to confirm clonal transmission, as isolates that display identical or highly related STR profiles with only 1 marker difference, might still originate from the same source within a hospital, since STRs are highly variable [38]. In addition, the epidemiological situation of the regions needs to considered. In a region with low prevalence, an identical or highly related STR profile is much more likely to indicate clonal transmission than a region with high prevalence. In India *C. auris* is currently highly prevalent and it was the first country to report a *C. auris* outbreak in 2012 [44]. Application of STR typing to clinical and environmental isolates collected from 2019 to 2020 from a single chest hospital in Delhi identified multiple clade I genotypes closely related to each other [45]. Here the closely related STR genotypes indicated multiple strains circulating within the hospital, as confirmed by WGS SNP analysis. Thus when the typed species is highly prevalent and relatedness is suspected based on STR typing, WGS is required, while in a region with a low prevalence the chance of two independent introductions with an identical STR genotype is low and STR analysis is sufficient to point out clonal transmission [46].

### *Candida parapsilosis*

Globally, but especially in southern Europe and Türkiye, *C. parapsilosis* is a common diploid pathogen in healthcare settings with emerging resistance to azoles and echinocandins and the cause of numerous outbreaks [47, 48]. An STR assay developed by Sabino et al*.* [49] used initially 11 markers but only retained three markers, amplified in monoplex, which showed the highest discriminatory power in a global collection (Table 1). By performing WGS SNP calling on isolates with identical STR genotypes according to the scheme of Sabino et al*.* [36] isolates displayed a low genetic difference. A subsequent study of Diab-Elschahawi et al*.* [50] added three markers in multiplex to the previous three markers. Using these STR assays, *C. parapsilosis* clusters were found that occasionally consist of fluconazole resistant isolates sharing the same resistance mechanism [50–52]. Many of these clusters were not restricted to a single ward, highlighting the ability of *C. parapsilosis* to cause widespread transmission when infection control measures are not properly enforced [53]. More recently, STR typing of the scheme by Diab-Elschahawi et al*.* [54] was applied to isolates from an Italian center, also uncovering large clusters. In another study, STR results were compared to FTIR outcomes [54]. The genotyping results did not correlate well, suggesting the accuracy of FTIR requires improvement, as it was previously shown that STR results correlate well to WGS. Due to the good correlation with WGS and high resolution, STR typing of *C. parapsilosis* is highly suitable to investigate ongoing outbreaks [51, 55].

### *Candida tropicalis*

The diploid *C. tropicalis* is considered one of the major medically important yeast species, also due to its increasing azole resistance, and is especially prevalent in tropical regions, like Latin America and India [32]. To date, three STR schemes were developed that amplify different markers (Table 1) [56–58]. The first reported scheme from Wu et al*.* [56] used six markers in monoplex and typed a set of Chinese isolates with both MLST and STR. Loci were identified from a single reference genome. As expected, STR analysis generated more genotypes than MLST, and as such MLST clusters were further differentiated in related STR genotypes. Application of this STR scheme pinpointed the gastrointestinal tract as the source of invasive *C. tropicalis* infections as isolates from this source had identical genotypes when compared to isolates obtained from blood [35]. The second scheme of Fan et al*.* [58] consists of eight markers, extracted from one reference genome, amplified in monoplex and was also applied to Chinese isolates. Here, STR results were compared to pulsed-field gel electrophoreses (PFGE), which compares chromosomal bands separated by gel electrophorese, with PFGE having a slightly higher discriminatory power, but has a long turnaround time and poor inter-lab comparability [11, 58]. Both methods correlated well in this study with the STR scheme slightly less discriminatory. The most recent developed STR assay of Spruijtenburg et al*.* [57] consists of six markers, is amplified in two multiplex PCR reactions and was developed using one reference genome and five unrelated isolates to identify conserved flanking regions. Comparison to the gold standard WGS demonstrated that the number of different microsatellite markers correlated well with SNP differences, while two isolates with an identical STR profile differed only 111 SNPs. Application of this scheme led to the identification of clusters and related isolates enriched for azole resistance in Brazil and Egypt, which also shared the same *ERG11* substitutions [59, 60]. Based on the, at present, limited comparison to WGS, it is not possible to delineate the discriminatory power of these three STR schemes. Ideally, the resolution of these schemes should be compared by applying them on a set of isolates also typed by WGS SNP analysis. While most STR markers are selected based on their high variability because of the high mutation rate, it is worthwhile to hypothesize that for individual markers a lower variability might be beneficial to establish the clade origin. For *C. tropicalis* a recent genotyping study using WGS and MLST on a large global collection demonstrated different clades, of which some were enriched for azole resistance [61]. The present STR schemes are unable to allocate isolates to a specific clade, as most markers are too variable and their copy number is not unique for a specific clade. Markers with long repeat units are generally more conserved and are more likely to provide unique genotypes for a specific clade [38]. Such markers might be added to current schemes to allocate isolates to clades. Overall, all STR genotyping assays applied to *C. tropicalis* showed a high genetic diversity with limited nosocomial transmission and rarely outbreaks involved [59, 62].

### *Candida glabrata*

In Europe and the United States, *C. glabrata* (also known as *Nakaseomyces glabratus*) is the second yeast species causing candidemia after *C. albicans*. *C. glabrata* exhibits intrinsically elevated minimum inhibitory concentrations (MICs) for fluconazole and is haploid [32]. One STR scheme was developed with the use of a single reference genome and eight markers were amplified in monoplex on a global isolate collection (Table 1) [63]. Subsequent application on isolates from the gastrointestinal tract and blood from French patients found clustering of isolates from these different sampling locations within single patients [64]. As *C. glabrata* is a commensal species in the gastrointestinal tract, it is thought that colonized patients can developed deep infections when the immunological status of patients decreases [64]. Other STR studies reported comparable results, with an overall high genetic diversity between isolates, indicating limited clonal transmission between patients [65, 66]. To date, no comparison between STR and WGS results have been made which is required to estimate the discriminatory power.

### *Candida krusei*

The last *Candida* species listed on the WHO Fungal Priority Pathogens List [5], *Candida krusei*, also known as *Pichia kudriavzevii*, is often diploid and sometimes triploid [67]. Fluconazole prophylaxis is a known risk factor as the species has intrinsic elevated MICs to this antifungal. To date, two typing assays have been developed (Table 1) [28, 68]. The first scheme of Gong et al. [68] selected 33 loci extracted from a single reference genome. These were successfully amplified in monoplex for 48 clinical Chinese isolates from 15 hospitals in ten cities. All isolates were differentiated from each other [68]. For future application, the authors suggested only eight loci to retain the discriminatory power while reducing costs and turnaround time. Further reduction of markers might be possible, as application of three out of eight final markers of the scheme from Gong et al*.* [68] was also sufficient to differentiate all isolates, although additional markers will likely increase the resolution when typing larger isolate collections. The scheme of van Haren et al. consists of six markers amplified by two multiplex PCRs. Conserved primer binding sites were identified with WGS of five geographically diverse isolates and all markers were subsequently successful amplified for a global isolate collection, showing a high genetic diversity. Application of this scheme identified clusters suggesting nosocomial transmission, which was subsequently confirmed with WGS for some isolates [28]. More recently, the scheme was used to type *C. krusei* isolates causing candidemia and vulvovaginal candidiasis from Türkiye and all isolates displayed unique genotypes, suggesting nosocomial transmission is an overall rare event for this species, as was previously also indicated with MLST [69, 70]. A comparative study of both STR schemes and WGS investigating the same isolates would establish the discriminatory power of both assays.

### *Wickerhamomyces anomalus*

In addition to the six WHO yeast priority pathogens [5], assays for rare species were developed as well. *Wickerhamomyces anomalus*, previously known as *Candida pelliculosa* or *Pichia anomala*, is diploid and frequently used in the food industry for fermentation purposes [71]. On rare occasions, this yeast is able to cause invasive bloodstream infections, with immunocompromised neonates at highest risk [72]. A single STR scheme was developed and compared to WGS results (Table 1) [73]. Markers and conserved flanking regions were selected based on WGS data of five geographically diverse isolates, leading to the inclusion of six markers in two multiplex PCRs. The analysis of some isolates by WGS demonstrated that most isolates with an identical STR genotype exhibited exceptionally low SNP numbers with eight SNPs at most [73]. The scheme was applied to a large collection of mainly Indian isolates and four large clusters were found in a single hospital, with clonal transmission of multiple strains concurrently taking place in multiple wards. Remarkably, one isolate showed an unexpected high number of 210 SNPs when compared to the other isolates with an identical STR genotype. Upon closer visual inspection of WGS data, nearly all SNPs were allocated to a region of approximately 400 kb in one chromosome. The other isolates were heterozygous in this region while the deviant isolate was homozygous. This difference is likely explained by loss of heterozygosity (LOH). LOH can be induced in a short timeframe by different forms of stress that include azole exposure and high temperatures [74]. As such, LOH can result in incorrect inferred genetic relatedness with automated SNP calling pipelines when SNPs are not visually inspected. At the moment, the extend of LOH occurring during outbreak situations is unknown but further investigation is warranted for diploid species.

### *Candida orthopsilosis*

*C. parapsilosis* and the rare pathogens *C. metapsilosis* and *C. orthopsilosis* are members of the diploid *C. parapsilosis* species complex [75]. For *C. orthopsilosis*, an STR typing assay was recently developed based on an initial number of 51 loci present in three reference genomes (Table 1) [76]. Subsequent application of all 4 markers in monoplex on 68 Chinese isolates, which included invasive isolates, uncovered two large clusters. These clusters were also allocated to the same AFLP and ITS genotype, although the overall discriminatory power of STR was higher in this study. The authors suggested using only six out of the 51 markers to achieve the same discriminatory power [76]. Given that most *C. orthopsilosis* are hybrid isolates that frequently undergo LOH [77], and LOH did not alter the STR genotyping results of *W. anomalus*, STR might pose a more suitable typing option if WGS SNP results are automated or not carefully inspected.

### *Kodamaea ohmeri*

Another rare human haploid pathogen is *Kodamaea ohmeri* (former *Pichia ohmeri*), for which infections are globally reported, albeit sporadically. This yeast is commonly used in the food industry for fermentation [78]. Recently, a typing scheme consisting of three markers that were selected from 50 loci present in one reference assembly and amplified in monoplex, was applied to a relatively large collection of Chinese isolates collected from 30 hospitals (Table 1) [79]. Multiple clusters were found that were mostly restricted to a single center involving multiple hospital wards. Additionally, one cluster was found to be fluconazole resistant. This indicates multiple events of nosocomial transmissions of either susceptible or resistant isolates. A notable downside of this scheme is the limited number of markers amplified. Although this reduces costs, the discriminatory power is likely lower in comparison to other STR schemes that include more markers. This remains to be investigated using a larger collection of genetically different isolates and comparison to WGS data.

#### *Diutina catenulata*

The rare opportunist yeast *Diutina catenulata*, previously known as *Candida catenulata* causes fungemia in immunocompromised hosts [80]. Furthermore, acquired azole resistance via mutations in *ERG11* has been reported [80]. A recently developed STR scheme with four markers extracted from a single reference genome was applied on 45 isolates in monoplex (Table 1) [81]. A total of 22 genotypes was observed. These grouped in eleven clades, often consisting of isolates from the same country. Three out of four isolates with mutations in *ERG11* clustered, suggesting a common ancestor acquired the mutation. Moreover, finding isolates with identical genotypes within a single hospital suggested clonal nosocomial transmission [81].

#### *Cryptococcus neoformans*

The basidiomycetous haploid yeast *C. neoformans* is an opportunistic pathogen in immunocompromised patients and mainly causes infections of the central nervous system, but may also present as pulmonary and/or disseminated disease [82]. Limited human-to-human transmission is reported and various environmental niches of the pathogen have been identified [82, 83]. The first scheme of Hanafy et al*.* [84] selected 15 random loci from one reference genome and amplified these markers in monoplex in a global isolate collection, resulting in three markers with the highest variability (Table 1). Application of this scheme on environmental and clinical isolates showed a clear distinction between the two sources that identified 13 genotypes in 53 environmental isolates that were mostly different from the ten genotypes found in 36 clinical isolates [85]. Interestingly, no difference in antifungal resistance or capsule size was observed suggesting another explanation for the source clustering. The second assay of Illnait-Zaragozi et al*.* [86] selected markers based on the same reference genome which resulted in amplification of nine markers in three multiplex PCRs, which was applied to a Cuban collection of 190 isolates identifying 109 genotypes (Table 1). Genetic diversity of 426 Asian isolates of *C. neoformans* was determined using the same STR analysis showing a correlation of the genotypes with the original source of the isolates and resistance to 5-flucytosine [87]. Other Dutch and Cuban studies on meningitis patients showed recurrent infections that were caused in some patients by the same strain while other patients acquired a new strain causing the infection [86, 88]. An Indian study showed a separation between clinical and environmental isolates but also compared the results to MLST showing a good correlation [89].

#### *Cryptococcus deneoformans*

*Cryptococcus deneoformans*, originally known as *C. neoformans* var. *neoformans* (serotype D), was classified as a distinct species via molecular phylogenetics [90]. Together with *C. neoformans*, these species are the most clinically relevant of the *C. neoformans* complex and are also frequently isolated from the environment [90]. In the aforementioned study on *C. neoformans* by Hagen et al*.* [88] an STR typing scheme for *C. deneoformans* was also developed. This scheme consists of seven loci found in a single reference genome and was species specific (Table 1). From 53 isolates 32 genotypes were found, while two distinct strains were found in one patient. Such coinfection was also found in a genotyping study on both *C. neoformans* and *C. deneoformans* isolates from Slovenia, which showed the presence of both species in some patients[91]. Finally, a single hybrid (serotype AD) was found with STR typing, highlighting the ability to resolve hybrid species and their haploid parentals.

#### *Cryptococcus deuterogattii*

While *C. deuterogattii,* previously known as *Cryptococcus gattii* AFLP6/VGII, infections are less common than *C. neoformans* and *C. deneoformans*, they have been isolated across wide geographic ranges and outbreaks have also been reported [92]. The most prominent outbreak occurred on Vancouver Island and the Pacific Northwest affecting healthy individuals [92]. A STR genotyping assay consisting of ten markers, amplified in three multiplex PCRs and one monoplex PCR, was applied to a global collection of 178 isolates (Table 1) [93]. STR results, supported by AFLP and MLST data, showed the highest genetic and recombination diversity in Amazonian isolates when compared to isolates from other continents, suggesting there was an ancient dispersal from the Amazon rainforest. This study also found that the notorious Vancouver Island outbreak was genetically unrelated to the Pacific Northwest outbreak, where STR greatly improved the understanding of *C. deuterogattii* outbreaks and dispersal [93].

## STR Typing of Medically Important Filamentous Fungi

### *Aspergillus fumigatus*

Due to its high prevalence in invasive and pulmonary aspergillosis, *A. fumigatus* was one of the first haploid fungal species for which STR schemes were developed (Table 2) [94, 95]. While the scheme of Bart-Delabesse et al*.* applied four markers in monoplex, the scheme of de Valk et al*.* [94, 95] called STR*Af* utilizes nine markers amplified in three multiplex PCRs. For both assays the markers were selected based on a single reference genome, possibly limiting amplification of genetically divergent genotypes harboring mutations in the primer binding sites. Phylogenetic investigations by both schemes showed high genetic diversity with clinical and environmental isolates randomly distributed, indicating likely exchange between environmental and clinical isolates [96–98]. The STR assay of de Valk et al*.* [99–101] was used to investigate the increasing number of *A. fumigatus* aspergillosis cases within single healthcare centers. This revealed an absence of large clusters, indicating limited clonal propagation within these centers, which was confirmed by WGS in one study [99]. The STR of de Valk et al*.* [102] was also compared with AFLP, which demonstrated a higher discriminatory power for STR to genotype *A. fumigatus*. This allowed the identification of multiple genotypes in respiratory samples with the same STR assay while these were not found with AFLP [102]. Lastly, in another study, the STR genotype of *A. fumigatus* was successfully determined in formalin-fixed paraffin embedded tissues and serum samples from five patients with invasive aspergillosis [103].

**Table 2:** Overview of short tandem repeat (STR) scheme developed for filamentous and other fungi. For each scheme, the number of developed assays, amplified markers and validation with whole genome sequencing (WGS) is specified

Species	No. of assays	No. of markers	PCR characteristic	Isolate application	WGS validation (no. of isolates)	References
*Aspergillus fumigatus*	2	4	Monoplex	Global	No	[94]
		9	Multiplex (3)	Global	Yes (14)	[95]
*Aspergillus flavus*	3	7	Multiplex	French and Tunisian	No	[107]
		24	Multiplex	American	No	[108]
		9	Multiplex (3)	Indian, Iranian	Yes (11)	[106]
*Aspergillus terreus*	1	9	Multiplex (3)	Global	No	[113]
*Microsporum canis*	3	2	Monoplex	Global	No	[117]
		8	Monoplex	Global	No	[119]
		6	Multiplex (1)	USA	No	[120]
*Sporothrix brasiliensis*	1	9	Multiplex (3)	Brazilian	Yes (21)	[125]
*Sporothrix* species*	1	15	Multiplex (5)	Brazilian	No	[124]
*Exophiala dermatitidis*	1	6	Multiplex (2)	Global	No	[128]
*Pneumocystis jirovecii*	1	6	Monoplex	Global	No	[133]

*This STR scheme includes *Sporothrix brasiliensis, S. schenckii* and *S. globosa*

To date, *A. fumigatus* is the only fungal species for which an allelic ladder has been developed, allowing direct comparison of STR results between laboratories [104]. The ladder consists of reference fragments of varying sizing that serve as a calibration for the gel electrophoresis. Five laboratories with experience in STR typing all typed the same isolates and found that the length of amplified markers can differ up to nearly seven nucleotides [104]. With the use of the allelic ladder, all laboratories were able to produce identical copy numbers, which could be stored in a global genotyping database as is common practice for MLST.

### *Aspergillus flavus*

In medicine and agriculture, the haploid species *A. flavus* is highly relevant as it is a common agent of invasive aspergillosis and may produce aflatoxins that contaminate crops [105]. A total of three STR assays were developed of which two were applied to mainly clinical isolates and one exclusively to environmental isolates (Table 2) [106–108]. The environmental scheme of Grubisha et al*.* [108] consists of 24 loci that were identified in one reference genome and are amplified in multiplex. The STR scheme by Rudramurthy et al*.* utilizes nine markers based on a single reference genome, which are amplified in three multiplex PCRs. This assay was applied to an Indian collection with various clinical presentations, yielding a high genetic diversity as was also found with AFLP [106]. However, with STR multiple genotypes were found in clinical samples, indicating the presence of distinct strains and demonstrating the inferior discriminatory power of AFLP [106]. Some isolates from different patients had identical genotypes, suggesting clonal expansion within the hospital environment, with construction work or air filtering systems as a potential source comparable to what was found for *A. fumigatus* [99]. The same microsatellite typing scheme of 143 clinical and environmental Iranian isolates demonstrated 118 different genotypes with a high diversity index with identification of a possible outbreak at a pulmonary ward [109]. STR and WGS was used to characterize a series of 11 isogenic *A. flavus* isolates isolated from a patient with pulmonary aspergillosis. Over a period of three months, the initially azole-susceptible isolate developed azole resistance. STR analysis and WGS revealed high genetic relatedness of all isolates, indicating a persistent infection by one single genotype [110]. Another scheme of Hadrich et al*.* [107] identified again the repeats from one reference genome that utilized seven markers in multiplex. The scheme was applied to isolates from France and Tunesia and found a high degree of genetic diversity and geographical clustering with no indication of nosocomial transmission. Application of this assay on a total of 29 environmental and clinical avian isolates demonstrated unique genotypes of all isolates, except for one clinical and one environmental isolate that shared an identical genotype [111].

### *Aspergillus terreus*

The proportion of *A. terreus* in clinical specimens is generally low, with an increase in specific regions [112]. Yet, the haploid pathogen is widely studied as it has intrinsically elevated MICs to amphotericin B, which correlates with clinical failure [112]. A single STR assay has been developed to date, which amplifies nine markers in three multiplex PCRs [113]. The markers were identified from a single reference genome and primers were designed on flanking sequences without the use of WGS to identify conserved regions. Multiple studies indicated a high genetic diversity with the identification of many unique genotypes as was also found with AFLP (Table 2) [113]. In a single patient, multiple isolates were found to be genetically distinct, indicating colonization by multiple strains [113]. No clustering based on geographic region or clinical presentation was found in two studies from France and India [113, 114]. Conversely in Tyrol, Austria, dominant genotypes from clinical samples and the environment were found [115]. This suggests patients acquire a clonal propagating strain from the environment, but it remains to be determined what the source in this region is.

### *Microsporum canis*

The zoophilic dermatophyte *Microsporum canis* is globally distributed but the epidemiology can differ based on age or sex [116]. Transmission either occurs by contact with infected animals or via humans. Three microsatellite typing schemes have been developed to study the genetic relatedness and transmission of isolates [116]. The scheme developed by Sharma et al*.* [117] included two dinucleotide repeats that were amplified in monoplex in 101 M*. canis* isolates from various continents (Table 2). Genotyping resulted in 11 genotypes with the largest cluster consisting of 50 isolates. Subsequent application by da Costa et al*.* on a total of 102 human, feline and canine *M. canis* isolates from Brazil found 14 genotypes without the presence of the dominant genotype found earlier by Sharma et al. [117, 118]. Another scheme was developed by Pasquetti et al*.* and involved six dinucleotide repeats identified from a single reference genome, in addition to the two markers from Sharma et al*.* [119] there were all amplified in monoplex (Table 2). Genotyping of 26 isolates revealed eight genotypes, indicating a higher discriminatory power than previous studies employing only two markers. A subsequent study from the USA genotyped 180 isolates used the STR scheme of Pasquetti et al*.* [120] and found 122 unique genotypes with two feline clusters restricted to two states, indicating a shared source. Different Japanese studies also utilized the scheme of Pasquetti et al*.* [116, 121] and found few isolates displaying identical genotypes although one notable example was a cluster within a family with a domestic cat as the possible source of infection. Aneke et al*.* included three markers from Pasquetti et al*.* [122] and included three novel markers, which were all six combined into a single multiplex PCR, thereby reducing the costs and increasing typing capacity (Table 2). Typing of 66 isolates from southern Italy using this scheme found 18 genotypes that could be divided into two clusters, allowing the authors to compare genotypes to differences between hosts and their symptoms. Their scheme proved useful for genotyping, although no significant differences were found between clusters regarding symptoms and virulence.

## Other Medically Important Fungi

### *Sporothrix brasiliensis*

Within Latin America, the dimorphic haploid fungus *Sporothrix brasiliensis* is posing a major health threat, affecting large numbers of humans and cats [22]. Since the first retrospective report from Rio de Janeiro, Brazil in 1998, the prevalence is nearing epidemic proportions in some densely populated areas and is recently reported in neighboring countries, as well as in the United Kingdom [123]. Recently, two STR assays were developed with a different species selectivity. One scheme consists of 15 markers amplified in 5 multiplex PCRs and is able to type *S. brasiliensis*, in addition to the medically relevant and closely related *Sporothrix* species, *S. schenckii* and *S. globosa* (Table 2) [124]. The ability to type multiple species is unique among STR typing schemes, which are usually restricted to a single species. By applying this assay to a varied collection of Brazilian isolates, a high genetic diversity was found for all three species. Unfortunately, no comparison or validation with other genotyping methods was performed, so the discriminatory power of this assay remains to be determined. The other STR scheme was solely developed for *S. brasiliensis*, utilizes nine markers amplified in three multiplex PCR reactions, and was applied to a different collection of Brazilian isolates [125]. On a large subset of these isolates, WGS was performed to validate the inferred genetic relatedness and estimate the typing resolution. Isolates with an identical STR genotype differed less than 150 SNPs, a discriminatory power comparable to assays for other aforementioned species. Most interestingly, the results of both developed assays in combination with WGS clearly demonstrated that *S. brasiliensis* is not a clonal species as previously suspected. The observed genetic diversity could not have been accrued since its first report, but suggests that the species is introduced independently numerous times into the mammalian population in Brazil. In addition, large STR clusters spanning wide geographic areas were found, with isolates from both humans and cats, indicating zoonotic transmission. In Brazilian border countries, *S. brasiliensis* cases have recently been reported, and two human cases from Buenos Aires, Argentina were genotyped with the *S. brasiliensis* specific assay [126]. Both isolates were found to be identical, suggesting they originate from the same source and were found to be closely related to previously typed Brazilian isolates. Taken together, STR demonstrated to be a very powerful tool, greatly expanding the understanding of the population structure and transmission of *S. brasiliensis* in South America.

### *Exophiala dermatitidis*

The black haploid yeast-like fungus *E. dermatitis* is a ubiquitous fungus, frequently found in the respiratory tract of cystic fibrosis (CF) patients [127], and occasionally causes infections with various clinical presentations [128]. A recently established STR assay utilizing six markers, extracted from a single reference genome, in two multiplex PCR reactions, was applied to clinical and environmental isolates from various geographic regions (Table 2) [129]. Isolates were differentiated based on the source and region, with clinical isolates not found to be related to environmental ones. Additionally, the study investigated sequential isolates from CF patients and found persistent colonization of the same strain in some patients, while others harbored strains with different genotypes over time. With WGS it can be determined whether the inclusion of six markers yields sufficient discriminatory power for this haploid species.

### *Pneumocystis jirovecii*

The unculturable haploid *P. jirovecii* causes severe respiratory infections and detection depends on PCR or staining methods [130]. The pathogen poses a high transmission risk in immunocompromised patients, making genotyping techniques necessary to track the spread and epidemiology within healthcare centers, especially when there is a suspicion for outbreaks [10]. A microsatellite scheme that utilizes six markers, extracted from a single reference genome, was subsequently applied in monoplex to respiratory samples from two French hospitals and compared to a MLST scheme (Table 2) [131, 132]. Once again, STR found more genotypes than MLST. Another study conducted on samples from Uganda, Spain and the United States indicated high genetic diversity as well, and did not find any country specific clusters or clades [133].

## Future Directions

Since the first established fungal STR scheme two decades ago, the number of applications is steadily increasing and a multitude of medically important fungal species are now covered. STR typing is a high-resolution typing method, is highly reproducible, has a low turnaround time and is relatively easy to implement. As such, STR genotyping certainly has a role in at least mycology reference laboratories performing outbreak analyses. The interchangeability of typing results between laboratories would require more allelic ladders to be developed in order to create global databases as was done for MLST. Alternatively, reference isolates can be used to compare results between laboratories but it should be taken into account that copy numbers may change over time.

For most rare or novel species there are no STR typing schemes available but in these instances WGS would be a more suitable option as development of a specific STR scheme is time-consuming [134]. Future research could focus on the develop schemes for other (emerging) invasive species, but also non-invasive species like *Trichophyton indotineae,* as little phylogenetic studies have been performed for this emerging global threat [135]. Although most well-established laboratories have the equipment to perform STR typing, the costs associated with high resolution gel electrophoresis and PCR might still be too high for low income countries, which is highly unfortunate given that many outbreaks take place there. In the near future, a large panel of STR markers amplified with PCR followed by NGS to determine the copy number could replace gel electrophoresis. Long read protocols are currently in development for human STR typing which could be adapted to fungal species [136]. This would probably also allow the easy inclusion of more markers to be analyzed and compared, although setting up the NGS infrastructure is certainly a challenge.

Another future direction is the inclusion of less variable markers like was done for *C. auris* [30]. If only highly variable markers would be included, isolates can still be differentiated but cannot be assigned to the corresponding clade. For other species like *C. tropicalis*, WGS also demonstrated clades that could not be visualized using STR genotyping due to overly variable markers [57, 61]. With the inclusion of markers with more conserved long repeat units, it is possible to assign isolates to their corresponding clade. Lastly, we suggest a professional standard, that each newly developed STR scheme should include validation by WGS on both related and unrelated (global) isolates to estimate the discriminatory power, demonstrating its merit for (clinical) practice.
